# Maximise Your Marks: Examining Self-Assessment Portfolio Scoring in UK Higher Psychiatry Training Applications

**DOI:** 10.1192/bjo.2026.11168

**Published:** 2026-06-30

**Authors:** Eve Morrison, Rohan Krishnan, Natasha Hobbs-Lakin, Sitki Anil Ustun, Nermeen Ahmed

**Affiliations:** 1 South London and Maudsley NHS Foundation Trust, London, United Kingdom; 2 East London NHS Foundation Trust, London, United Kingdom; 3 Gloucester Health & Care NHS Foundation Trust, Gloucester, United Kingdom; 4 North East London NHS Foundation Trust, London, United Kingdom; 5 Greater Manchester Mental Health NHS Foundation Trust, Manchester, United Kingdom

## Abstract

**Aims:**

The national recruitment process for higher psychiatry training in the UK includes a self-assessment section that recognises applicants’ experience and achievements outside of their usual work. Evidence is uploaded by applicants then reviewed by verifiers using a standardised scoring system. The total awarded score ultimately influences applicants’ priority ranking when training offers are allocated. The aim of this study is to identify trends in self-assessment scoring inaccuracies and explore the reasons for these. This will inform future improvements to the self-assessment process and scoring guidance.

**Methods:**

Anonymised data was provided by the National Psychiatry Recruitment Office for all applicants to higher psychiatry training in February and August 2025. Counts were summarised overall and by group using descriptive statistics. Thematic analysis was conducted to explore reasons for scoring adjustments and appeals across the ten assessed domains. Coding was cross-checked by a second researcher to enhance validity.

**Results:**

There were 1,025 applicants across the two recruitment rounds, with 76% having completed core psychiatry training or being in their final year of training. Fifty-nine per cent of applicants were non-UK graduates. The findings highlighted frequent errors in both applicants’ evidence submissions and in verifiers’ reviews. Common applicant error themes included submitting inappropriate, excessive, or incomplete evidence, misunderstanding current guidance, and incorrect domain allocation. Upheld appeals highlighted several instances of incorrect verifier scoring due to misinterpretation of submitted evidence and verification guidance. This resulted in frequent misalignment between candidates’ self-assessments and verifiers’ scoring, with 16% of applicants appealing their scores. Most scoring errors were identified in domains 6 (Clinical Governance, Audit and Quality Improvement as a postgraduate), 8 (Teaching), and 10 (Presentations and Poster Presentations). Scoring errors disproportionately affected applicants with non-UK qualifications and those submitting evidence obtained abroad as documents often did not conform to UK formats, contributing to scoring misalignment.

**Conclusion:**

These findings highlight several challenges in translating applicants’ achievements into standardised scores in the current Higher Psychiatry Training recruitment process. Frequent scoring errors by both applicants and verifiers indicate that the current guidance may lack sufficient clarity, particularly regarding evidence requirements, domain boundaries and scoring thresholds. There is a need to develop the applicant and verifier guidance to include clearer definitions, descriptions, and examples to reduce error rates and improve equity and consistency in portfolio scoring.

